# ER Stress Is Associated with a “Mesenchymal Drift” in an Anaplastic Thyroid Carcinoma Cell Line

**DOI:** 10.3390/cancers17213534

**Published:** 2025-10-31

**Authors:** Dario Domenico Lofrumento, Alessandro Miraglia, Antonella Sonia Treglia, Francesco De Nuccio, Giuseppe Nicolardi, Corrado Garbi, Bruno Di Jeso

**Affiliations:** 1 Department of Biological and Environmental Sciences and Technologies (DiSTeBA), University of Salento, Ecotekne, Strada Monteroni, 73100 Lecce, Italy; 2 Department of Experimental Medicine, University of Salento, Ecotekne, Strada Monteroni, 73100 Lecce, Italy; alessandro.miraglia@unisalento.it (A.M.); sonia.treglia@unisalento.it (A.S.T.); francesco.denuccio@unisalento.it (F.D.N.); giuseppe.nicolardi@unisalento.it (G.N.); 3 Independent Researcher, 80100 Napoli, Italy; corgarbi@gmail.com

**Keywords:** anaplastic thyroid carcinoma, endoplasmic reticulum stress, cancer progression

## Abstract

**Background/Objectives:** The tumor microenvironment (TME) plays a crucial role in the progression of the malignant phenotype through several mechanisms, such as hypoxia and nutrient deprivation, among others. These insults activate several intracellular pathways, and among them are ER stress and the unfolded protein response (UPR). Our aim was to assess if a specific ER stress inducer causes an exacerbation of the malignant phenotype of anaplastic thyroid carcinoma (ATC) cells. **Methods:** We used an ATC cell line, FRO cells, that had not undergone a full Epithelial–Mesenchymal Transition (EMT) and an ER stress-adapted cell line derived from FRO cells, A400 cells. Western blot, immunofluorescence, scratch, and invasion assays were used to evaluate the response of the FRO and A400 cells to ER stress. **Results:** The FRO cells were subjected to high-level ER stress caused by 400 ng/mL of tunicamycin (Tn). This caused the death of a large fraction of cells, but eventually a population emerged that we called A400 cells. Following an over challenge with Tn, the adapted population showed suppression of the UPR, apoptosis, and stress kinase activation. Moreover, the adapted population showed an exacerbation of mesenchymal features with a more invasive phenotype. At the level of a single cell, the adapted cells, caught in the act of moving, showed high-level expressions of vimentin (VIM), fibronectin (FN), and N-cadherin. **Conclusions:** High-level ER stress acts as a selection factor favoring the emergence of a cell population showing “mesenchymal drift” with a more malignant phenotype.

## Simple Summary

A particular type of cellular stress that hits the endoplasmic reticulum (ER) may cause death or survival, depending on several factors, one of which is the intensity of the stress. However, data obtained by our group and others on normal and differentiated cells show that survival may be achieved at the expense of differentiation and, in thyroid epithelial cells, thyroid-specific proteins disappear, while mesenchymal markers appear in a variable number and extent, causing a “mesenchymal shift”. We hypothesize that these findings may be even more significant in a cancer context, where ER stress is linked to an exacerbation of the malignant properties of cancer cells, a process known as cancer progression. Here, we show that anaplastic thyroid cancer cells subjected to ER stress display an exacerbation of the malignant phenotype, with increased single-cell motility, invasion, and high-level mesenchymal protein expression in the moving component that has lost cell–cell contacts, features that may be defined as “mesenchymal drift”.

## 1. Introduction

Anaplastic thyroid carcinoma (ATC), albeit not common, represents one of the most lethal human cancers [1]. About 50% of patients show metastasis to distant organs at the time of diagnosis [2] and suffer extremely poor prognoses. Therefore, understanding the cellular and molecular mechanisms of ATC invasion and metastasis is critical for developing new strategies to improve the prognosis and survival rates for patients with ATC. The tremendous malignancy of ATC also demands increasingly accurate diagnosis. Therefore, firstly, the American Thyroid Association’s guidelines have recently become more stringent, suggesting that even 5 to 10 mm nodules with suspicious ultrasound features (hypoechogenicity, microcalcifications, infiltrative margins, and a taller-than-wide shape) should undergo Fine-Needle Aspiration Cytology (FNAC) because a subset of these behaves aggressively [3]. Secondly, the utilization of transcriptional signatures and discovery of driver mutations promoting thyroid cancer development and malignancy, improved diagnostic accuracy, and, recently, next-generation sequencing and micro-RNA technology platforms, have increased the diagnostic capacity of thyroid nodules [4].

Many cancers show, over time, an intensification of the malignant phenotype. Cancer cells both improve their capability to survive adverse conditions and become progressively more motile, invasive, and metastasizing through a process known as cancer progression. The tumor microenvironment (TME) plays a crucial role in the progression of the malignant phenotype. Intratumoral hypoxia leads to increased activity of hypoxia-inducible factors (HIFs) [5,6]. Over 1500 HIF target genes have been identified [5,6,7]. They contribute to angiogenesis, EMT, motility, invasion, metastasis, and resistance to chemotherapy. Moreover, solid tumors are composed not only of cancer cells but also of host-resident and recruited cells that interact and crosstalk, often driving cancer progression [8]. This is the case with the M2 alternative activation of tumor-associated macrophages, driven by IL-4 and IL-13 derived from TH2 cells [9], or basophils [10], which promote angiogenesis, specific immunity suppression, and cancer growth and metastasis [11]. In general, the TME perturbs ER homeostasis in infiltrating immune cells, altering their metabolism, differentiation, effector capacity, and antitumor activity, ultimately favoring cancer cell immune evasion, resistance to immunotherapy, and disease progression [12,13].

Thus, the TME promotes ER stress and activates the UPR [14,15]. In mammalian cells, the ER stress is sensed by three key ER resident transmembrane proteins, PRKR-like ER kinase (PERK), inositol-requiring enzyme 1α (IRE1α), and activating transcription factor 6 (ATF6) [16]. PERK is a protein kinase that, with other stress kinases, phosphorylates the alpha subunit of the cytosolic eukaryotic translation initiation factor eIF2, resulting in the inhibition of protein synthesis [17]. One consequence of eIF2α phosphorylation is the selective translation of certain mRNA, like that of transcription factor ATF4, which activates genes involved in amino acid biosynthesis and redox balance. IRE1 activates endoribonuclease activity in the cytosolic domain of the protein, leading to the unconventional splicing of the mRNA of a single gene, Xbp1 [18]. This splicing produces an active XBP1 transcription factor that controls the induction of a wide spectrum of UPR-related genes involved in protein folding, ERAD, cotranslational translocation, and quality control. ATF6 is a member of a transmembrane protein family cleaved by S1P and S2P to liberate active cytosolic transcription factors in response to ER stress [19]. Active ATF6 activates the transcription of target genes related to folding and ERAD, among others.

TME triggers ER stress in multiple ways. Tumor ischemia is one, because protein folding in the ER requires oxygen as the final acceptor of electrons [20]. The UPR is also activated via nutrient deprivation, since protein folding requires ATP and is sensitive to reduced intracellular glucose [21]. Low local extracellular pH, caused by cell aerobic glycolysis, causes ER stress in an ROS-dependent manner [22]. Limited glucose also results in sarcoplasmic/ER calcium ATPase (SERCA) activity inhibition and altered calcium homeostasis in the ER [23].

UPR leads to the activation of both adaptive and pro-apoptotic pathways. When ER stress is excessive or prolonged and recovery fails, the UPR ends with apoptotic cell death [24,25,26]. Recently, we and others have shown, using non-transformed cells, that the UPR does not imply a simple binary outcome, adaptation/survival, or death, but that a third alternative is possible: cells survive but differentiation is compromised [27,28,29] and mesenchymal features appear in variable numbers [30,31]. We call these phenotype changes a “mesenchymal shift”.

In this study, we sought to extend our previous results [31] to cancer cells and demonstrate that the adaptation of ATC cells to ER stress enhances not only cell survival but also mesenchymal characteristics, resulting in a “mesenchymal drift”. This may have important consequences on the strategy used to exploit the UPR in antitumor therapies, i.e., through inhibition of survival/EMT-like pathways.

## 2. Materials and Methods

### 2.1. Materials

All reagents were obtained from Sigma-Aldrich (Merk Life Science S.r.l., Milano, Italy) unless indicated otherwise. The primary antibodies were anti-Bip, anti-CHOP, anti-phosphorylated-eIF2α (P-eIF2α), anti-eIF2α, anti-phosphorylated-ERK (P-ERK), anti-ERK, anti-phosphorylated-JNK (P-JNK), anti-JNK, anti-phosphorylated-p38 (P-p38), anti-p38, anti-caspase 9, anti-PARP, anti-E-cadherin, anti-N-cadherin, anti-fibronectin (FN), and anti-β-catenin (Cell Signaling Technology, Danvers, MA, USA); anti-β-actin (Sigma-Aldrich); and anti-vimentin (VIM) (Merck Millipore, Merk Life Science S.r.l., Milano, Italy). The anti-rabbit/anti-mouse horseradish peroxidase-conjugated antibodies were obtained from Sigma-Aldrich.

### 2.2. Cell Culture and Selection Procedure

FRO [32], CAL62 [33], and 8505C [34] are cell lines derived from human ATC and were provided by Dr. A. Fusco. The TAD-2 cell line, obtained through Simian virus 40 infection of human fetal thyroid cells, was a gift from Dr. M. Illario. These cells were cultured in DMEM high-glucose medium supplemented with 10% fetal bovine serum (FBS). After the selection in Tn, 400 ng/mL A400 and FRO cells were grown in the same medium with the addition of Tn 400 ng/mL or vehicle, respectively. The selection was conducted in 100 mm diameter dishes with cells plated at 60% confluency in DMEM high-glucose medium supplemented with 10% FBS. After 48 h, 400 ng/mL Tn was added to the same medium. The Tn-added medium was changed every 3 days.

### 2.3. Western Blot Analysis

Tn and vehicle were removed from the culture medium 48 h before the experiment (in A400 and FRO cells, respectively). Then, 800 ng/mL Tn was added, and both cell lines were harvested at various time points. Western blots were carried out as previously reported [35,36]. Briefly, after evaluating the protein content, 30 μg of cell extract was analyzed via SDS-PAGE and electro-transferred to polyvinylidene difluoride. Blocking was performed for 15 h at 4 °C with Tris-buffered saline-Tween 20 (TBST) buffer (10 mM Tris [pH 8.0], 150 mM NaCl, 0.1% Tween 20) containing 10% nonfat dry milk, followed by incubation in TBST buffer for 2 h at room temperature with 1:500 dilution of anti-β-actin, and anti-P-ERK; 1:1000 dilution of anti-Bip, anti-ERK, anti-P-JNK, anti-JNK, anti-P-p38, anti-p38, anti-eIF2α, anti-P-eIF2α, anti-PARP, and anti-caspase 9; and 1:750 dilution of anti-CHOP. After being washed with TBST, the blot was incubated for 1 h at room temperature with the appropriate antirabbit or anti-mouse horseradish peroxidase-conjugated antibodies diluted 1:3000 in TBST. Bands were detected via enhanced chemiluminescence and autoradiography or using a ChemiDoc MP Imaging System (Bio-Rad S.r.l., Milano, Italy) for BiP Western Blot. Molecular mass markers were obtained from Bio Rad.

### 2.4. Immunofluorescence

A total of 1.5 × 10^5^ cells were plated on 12 mm diameter glass coverslips and grown to sub-confluence in DMEM high-glucose 10% FBS, Tn 400 ng/mL (A400 cells), or vehicle (FRO cells). Then, cells were incubated in DMEM high-glucose medium without FBS, Tn, or vehicle for 48 h. Cells were fixed for 20 min with 3% paraformaldehyde (Sigma) in PBS containing 0.9 mM CaCl_2_ and 0.5 mM MgCl_2_ (PBS-CM) at room temperature, and washed twice in 50 mM NH_4_Cl in PBS-CM and twice in PBS-CM. Cells were permeabilized for 5 min in 0.5% Triton-X 100 (Bio-Rad) in PBS-CM and incubated for 30 min in 0.5% gelatin (Sigma) in PBS-CM. Cells were then incubated for 1 h with the primary antibodies (anti-E-cadherin 1:100, anti-N-cadherin 1:100) diluted in 0.5% BSA (Sigma) in PBS. After three washes with 0.2% gelatin in PBS-CM, cells were incubated with the second primary antibodies (anti-β-catenin 1:100) for 1 h. After three washes with 0.2% gelatin in PBS-CM, cells were incubated for 20 min with the appropriate rhodamine- or fluorescein-tagged goat antimouse or antirabbit antibody (Jackson ImmunoResearch, West Grove, PA, USA), diluted 1:50 in 0.5% BSA in PBS. After final washes with PBS, the coverslips were mounted on a microscope slide and examined with a Zeiss 510 confocal laser scanning microscope (Zeiss S.p.a., Milano, Italy). Samples were observed by three investigators, without knowledge of the experimental conditions.

### 2.5. Scratch Wound-Healing Assay Coupled with Immunofluorescence

A total of 1.5 × 10^5^ cells were plated on 12 mm diameter glass coverslips and grown to sub-confluence in DMEM high-glucose 10% FBS with Tn (A400 cells), or vehicle (FRO cells). Then, cells were incubated in DMEM without FBS, Tn, or vehicle for 48 h. The cell monolayers were scratched with a sterile 200 μL pipette tip to create a wound, and cells were washed twice with serum-free DMEM high-glucose medium to remove floating cells, and then placed in medium without FBS, Tn, or vehicle. After 14–16 h, cells were processed for immunofluorescence, as described above.

### 2.6. Adhesion on Matrigel

To determine cell adhesion on Matrigel, 24-well plates were coated with Matrigel (diluted to 1 μg/mL in serum-free DMEM). A total of 10^5^ cells/well, pre-incubated in Tn-, vehicle-free DMEM high-glucose 10% FBS for 48 h, were then plated in serum-, Tn-, vehicle-free DMEM. Cells were incubated for 3 h at 37 °C. After 2 washes with PBS-CM, adherent cells were photographed under phase contrast at 10× magnification.

### 2.7. Transwell Invasion Assays

Cells were pre-incubated in serum-, Tn-, vehicle-free DMEM for 48 h. In vitro Transwell invasion assays were performed in Boyden chambers with 8 µm pore filter inserts in 24-well plates (BD BioCoat Matrigel invasion chambers, BD Biosciences, San Jose, CA, USA). The filters were pre-coated with 100 µL Matrigel diluted at 1 μg/mL in serum-free DMEM to form a reconstituted basement membrane. The lower chamber was filled with DMEM containing 10% FBS. Cells were collected after trypsinization, re-suspended in 200 µL of serum-, Tn-, vehicle- free DMEM medium, and transferred to the upper chamber (1 × 10^5^ cells/well). After 24 h of incubation, the filter was gently removed from the chamber, washed 2× with PBS-CM, and the cells on the upper surface were removed using a cotton swab. Cells on the lower surface were fixed (4% paraformaldeide in PBS-CM), permeabilized (Tritox-X100 0.2% in PBS-CM), and stained with hematoxilin. After three washes in PBS-CM, the cells were photographed.

## 3. Results

### 3.1. Isolation of “Selected/Adapted” FRO Population

**Figure 1:**
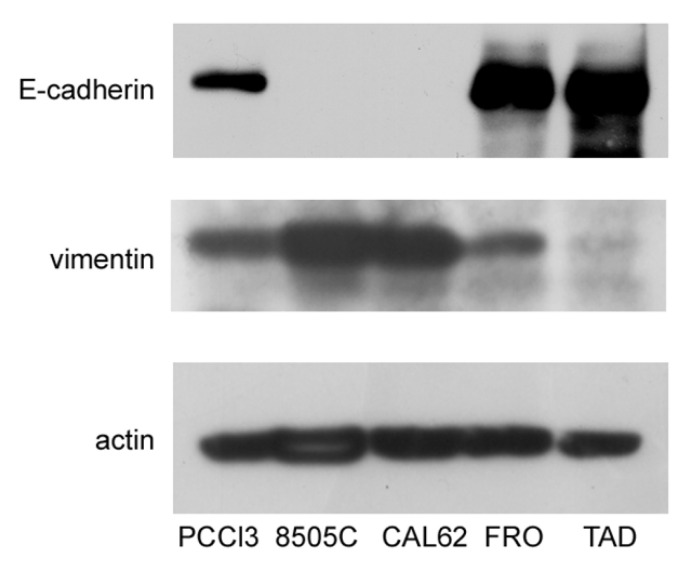
FRO cells have not undergone a full EMT. Cells were plated in 60 mm diameter plates to about 50% confluence 48 h before harvesting. Western blots of total protein extracts from PCCl3, 8505C, CAL62, FRO, and TAD cells were carried out as indicated in the Materials and Methods section. Uncropped Western blot images are shown in Supplementary Material Figure S3.

In this study, we sought to test the hypothesis that ER stress is associated with progression of the malignant phenotype of ATC cells. A preliminary issue was the selection of the ATC cell line, in that we needed cells that had not yet acquired a fully mesenchymal phenotype. We studied three ATC cell lines, FRO, CAL62, and 8505C. Cross-contamination, misidentification, and redundancy of known historical thyroid cancer cell lines have been reported [37]. However, the lines that we analyzed, FRO, CAL62, and 8505C, are unique and of thyroid origin [37]. 8505C [33] and FRO cells harbor the BRAF V600E mutation, whereas CAL62 cells exhibit the KRAS G12R mutation [33]. In addition, 8505C [34] and CAL62 [38] cells also have loss-of-function p53 mutations, while FRO cells have wildtype p53, although with decreased expression [32,39], suggesting that FRO cells retain a residual p53 function. These genetic differences suggest that FRO cells are at least a step behind, with respect to 8505C and CAL62 cells in the progression towards a more malignant phenotype. In addition to oncogene/oncosuppressor gene analysis, these cell lines have been extensively studied with regard to thyroid differentiation [37,40,41] and mesenchymal factors [42,43]. Next, we analyzed the expression of E-cadherin and vimentin in FRO, CAL62, and 8505C cells. As shown in Figure 1, while CAL62 and 8505C cells do not express E-cadherin, FRO cells do express it. Moreover, FRO cells express less vimentin than CAL62 and 8505C cells. These results are in line with the genetic differences between FRO cells, on the one hand, and CAL62 and 8505C cells, on the other hand.

**Figure 2:**
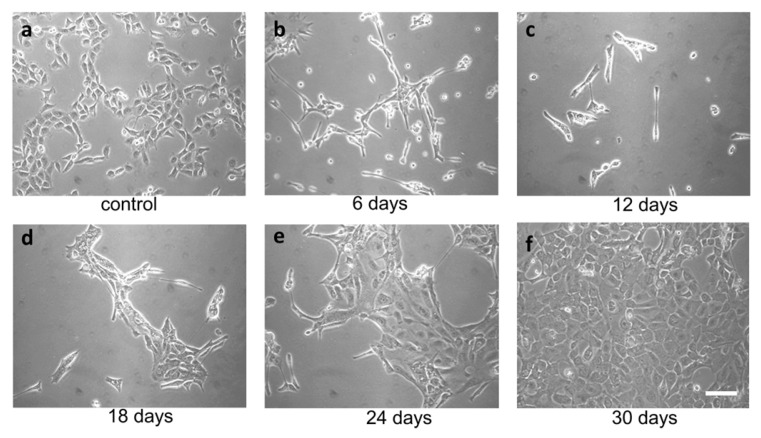
Selection of A400 cells. FRO cells routinely cultured in DMEM high-glucose medium supplemented with 10% FBS were plated at 60% confluency in 100 mmdiameter plates. After 48 h, 400 ng/mL Tn was added to the same medium for several days (panels (**a**–**f**)) and photographed. After extensive death, a population emerged that was able to grow in the presence of Tn 400 ng/mL (panels (**e**,**f**)) (A400 cells). Scale bar: 50 μm.

TME promotes ER stress, and UPR promotes tumor survival. In the context of a solid tumor, where oxygen and nutrients become limiting, cancer cells may experience strong ER stress. Therefore, to obtain a surrogate of the above situation, we treated FRO cells with a relatively high concentration of the ER stressor. We used Tn, which inhibits N-linked glycosylation in the ER, causing protein misfolding. Since N-linked glycosylation is an ER specific process, Tn is a quite specific inducer of ER stress. At 400 ng/mL, Tn caused the death of the majority of cells in a few days (Figure 2b,c), but then a population emerged (Figure 2d, called A400 cells) that was surprisingly able to grow in the continuous presence of Tn 400 ng/mL (Figure 2e) and could be grown for as long as their untreated counterparts (Figure 2f). Also, A400 cells appeared morphologically different, with increased size and decreased birifragency (Figure 2a vs. Figure 2e,f).

### 3.2. Attenuation of UPR Signaling in A400 Cells

**Figure 3:**
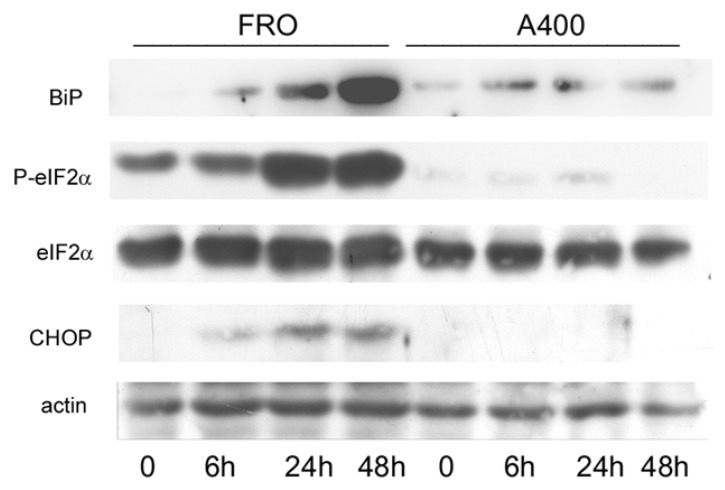
UPR signaling is attenuated in A400 cells. Cells were plated in 60 mm diameter plates to about 50% confluence in DMEM high-glucose 10% FBS with Tn (A400 cells) or vehicle (FRO cells). After 24 h, Tn and vehicle (in A400 and FRO cells, respectively) were removed from the culture medium for 48 h. Then, 800 ng/mL Tn was added various times to both cell lines. Western blots of total protein extracts from FRO and A400 cells were carried out as indicated in the Materials and Methods section. Uncropped Western blot images are shown in Supplementary Material Figure S4, and the densitometric analysis results are shown in Supplementary Material Figure S5.

Next, we compared the activation of the UPR in A400 cells with that of confluence-matched vehicle-treated controls (“naive cells”). Thus, FRO and A400 cells were challenged with 800 ng/mL Tn (a concentration twice that to which A400 cells were adapted). As shown in Figure 3, in A400 cells, BiP levels only modestly increased. In contrast, as expected, in “naïve” cells, BiP robustly increased. Moreover, A400 cells showed an absent induction of P-eIF2α and, accordingly, of CHOP, while in “naïve” FRO cells, both P-eIF2α and CHOP were potently induced (Figure 3).

**Figure 4:**
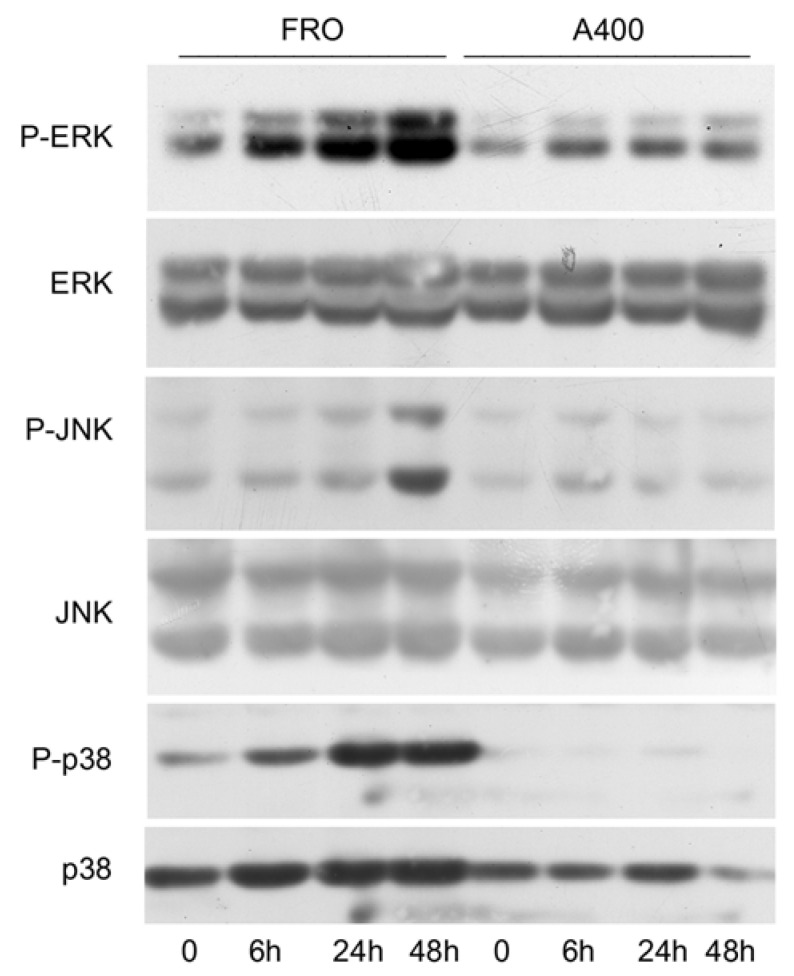
The activation of stress kinases is attenuated in A400 cells. Cells were plated in 60 mm diameter plates to about 50% confluence in DMEM high-glucose 10% FBS with Tn (A400 cells) or vehicle (FRO cells). After 24 h, Tn and vehicle (in A400 and FRO cells, respectively) were removed from the culture medium for 48 h. Then, 800 ng/mL Tn was added for various times to both cell lines. Western blots of total protein extracts from FRO and A400 cells were carried out as indicated in the Materials and Methods section. Uncropped Western blot images are shown in Supplementary Material Figure S6, and the densitometric analysis results are shown in Supplementary Material Figure S7.

Since it has been shown that signals emanating from the stressed ER, through IRE1, are able to activate the stress kinases, ERK, JNK, and p38 [44,45,46], we sought to verify if such a phenomenon will also be present in A400 cells. As shown in Figure 4, this was the case since in A400 cells the activation of ERK, JNK, and p38, following a 800 ng/mL Tn challenge, was absent or minimal, unlike with FRO cells, in which a robust Tn-induced activation of the stress kinases was present.

### 3.3. Apoptosis Is Suppressed in A400 Cells

The above results show that in selected/adapted cells, the UPR is attenuated. Since the UPR also leads to the activation of pro-apoptotic pathways, we studied cell survival following the same over-stimulation shown in Figure 3. While almost all FRO cells died on day 8 following a 800 ng/mL TN treatment, A400 cells survived on day 8 following the same treatment (Supplementary Figure S1a *iii* vs. *vi*). As anticipated by the lack of CHOP induction following a Tn challenge, A400 cells did not show either cleavage of the general apoptosis marker PARP or activation of caspase 9, unlike with FRO cells (Supplementary Figure S1b; uncropped Western blot images are shown in Supplementary Material Figure S8). Notably, cleaved PARP and caspase activation peaked at 24–48 h, synchronously with CHOP (Supplementary Figure S1b vs. Figure 3), which is a crucial apoptosis inducer of the UPR [47]. Although we cannot exclude activation of other death pathways, these data indicate that the apoptotic pathway was, indeed, activated in FRO cells and suppressed in A400 cells.

### 3.4. Analysis of Mesenchymal Proteins in A400 Cells

Taken together, Figure 3, Figure 4 and Figure S1 show that the cell population emerging from a strong ER stress acquires a growth advantage over parental cells, since activation of the UPR is attenuated and apoptosis is suppressed, allowing A400 cells to survive and grow in the presence of high Tn concentration.

We have shown that following ER stress and activation of UPR, normal epithelial thyroid cells, PC Cl3 cells, dedifferentiate, with downregulation of E-cadherin and upregulation of vimentin [31]. Thus, we wondered if the ER stress applied to cancer thyroid cells is followed by an exacerbation of the mesenchymal phenotype. 

We studied the cellular distribution of E-cadherin, N-cadherin, and β-catenin via double immunofluorescence assays. As shown in Supplementary Figure S2, in FRO cells, the E-cadherin signal overlapped quite well with the β-catenin signal at the plasma membrane (panel c, plasma membranes decorated in yellow). This overlap appeared more pronounced than the N-cadherin/β-catenin overlap (panel f, plasma membranes mostly in green). On the contrary, A400 cells showed prevalent N-cadherin/β-catenin overlap at the plasma membrane (panel n, plasma membranes mostly in yellow) with respect to the E-cadherin/β-catenin overlap (panel i, plasma membranes mostly in green). Moreover, while E-cadherin showed an evident intracellular pool in both cell types (panels b, c and h, i), N-cadherin still presented an intracellular pool in FRO cells (panel e, f), but it was almost exclusively confined to the plasma membrane in A400 cells (panel m, n). These results suggest a functional E-cadherin–N-cadherin switch in A400 cells.

### 3.5. Mesenchymal Proteins Were Preferentially Expressed in Migrating Cells

Next, we reasoned that analysis of epithelial and mesenchymal proteins could perhaps be better studied in dynamic conditions rather than in static ones, as shown above (Supplementary Figure S2). Thus, we sought to focus on moving cells rather than on cells “immobilized” in the monolayer. To translate this idea into an experimental protocol, we set up what, to the best of our knowledge, appears to be an original approach, a scratchwound migration assay coupled with immunofluorescence staining, to catch cells in the act of moving.

**Figure 5:**
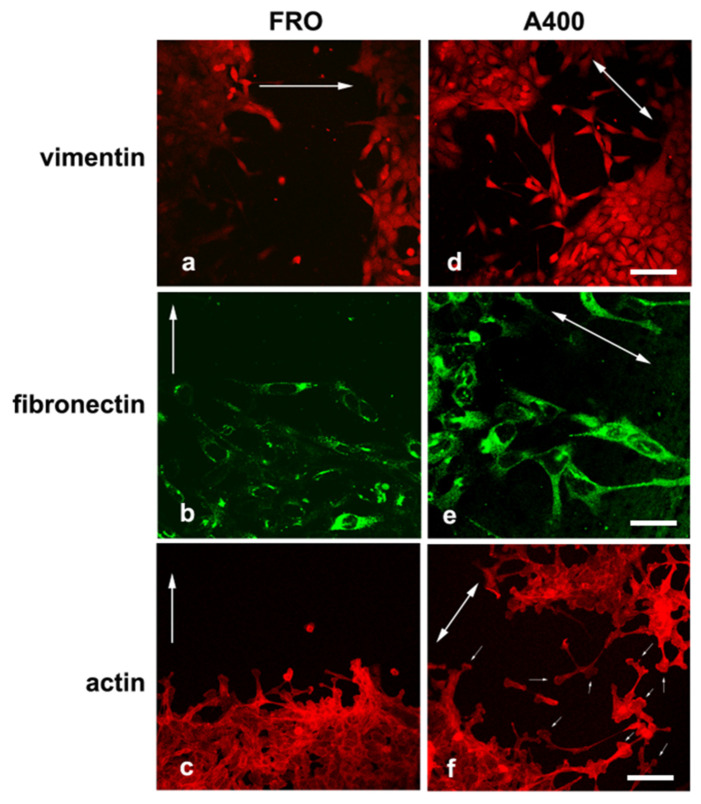
A400 cells, caught in the act of moving, are heavily stained with VIM and FN and show lamellypodia at the leading edge. Cells were plated on glass coverslips and grown to sub-confluence in DMEM high-glucose 10% FBS with Tn (A400 cells) or vehicle (FRO cells). Then, cells were incubated in DMEM without FBS, Tn, or vehicle for 48 h. The cell monolayers were scratched with a sterile 200 μL pipette tip to create a wound, washed twice with serum-free DMEM, and then placed in medium without FBS, Tn, or vehicle. After 14–16 h, cells were processed for immunofluorescence, as indicated in the Materials and Methods section. Panels (**a**–**c**): FRO cells immunostained with VIM, FN, and actin, respectively. Panels (**d**–**f**): A400 cells stained with VIM, FN, and actin, respectively. The big arrows indicate the direction of the movement; i.e., they are perpendicular to the major axis of the wound. The small arrows in panel (**f**) indicate lamellipodia at the leading edge of A400 cells. Scale bars: (**d**) 50 μm; (**e**) 10 μm; (**f**) 20 μm.

**Figure 6:**
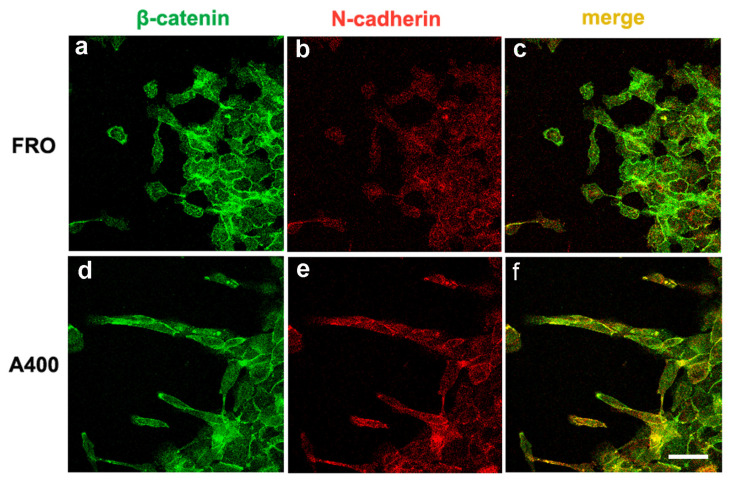
Migrating A400 cells were well stained with N-cadherin, mostly at the leading edge of the cells and strongly co-localizing with β-catenin. Cells were plated on glass coverslips and grown to sub-confluence in DMEM high-glucose 10% FBS with Tn (A400 cells) or vehicle (FRO cells). Then, cells were incubated in DMEM without FBS, Tn, or vehicle for 48 h. The cell monolayers were scratched with a sterile 200 μL pipette tip to create a wound, washed twice with serum-free DMEM, and then placed in medium without FBS, Tn, or vehicle. After 14–16h, cells were processed for immunofluorescence, as indicated in the Materials and Methods section. Panels (**a**,**b**): FRO cells immunostained with β-catenin and N-cadherin, respectively. Panel (**c**): β-catenin/N-cadherin overlay in FRO cells. Panels (**d**,**e**): A400 cells immunostained with β-catenin and N-cadherin, respectively. Panel (**f**): β-catenin/N-cadherin overlay in A400 cells. Scale bar: 20 μm.

We noted that A400 cells showed an increased tendency with respect to FRO cells to separate from the edge of the cell sheet and migrate into the free space created by the wound, as shown in Figure 5 (panels d, e, f vs. a, b, c). These cells were heavily stained with VIM (Figure 5d) and FN (Figure 5e). On the contrary, FRO cells largely failed to separate from the edge of the wound and were poorly stained with VIM (Figure 5a) and FN (Figure 5b). In addition, when an F-actin stain was performed, migrating A400 cells showed large lamellipodia at the leading edge (Figure 5f, small arrows), features that, although present in FRO cells, were confined to few cells at the migrating front of the edge (Figure 5c). Moreover, migrating A400 cells were well stained with N-cadherin, often present at the leading edge of the cells (Figure 6e) and strongly co-localized with β-catenin (Figure 6f). On the contrary, FRO cells, at the edge of the wound, were poorly stained with N-cadherin (Figure 6b), and there was no overlap with β-catenin staining (Figure 6c). These results show a propensity of A400 cells to separate from the monolayer and migrate into the free space, and that precisely these cells were heavily stained with FN, VIM, and N-cadherin, showing lamellipodia at the leading edge. Of note, when we measured the rate of migration of the fronts of the wounds, we did not obtain clear differences between FRO and A400 cells. Thus, it appears that A400 cells show increased solitary cell migration but not increased collective cell migration.

### 3.6. A400 Cells Displayed Increased Adhesion to Extracellular Matrix Proteins and Invasivity

**Figure 7:**
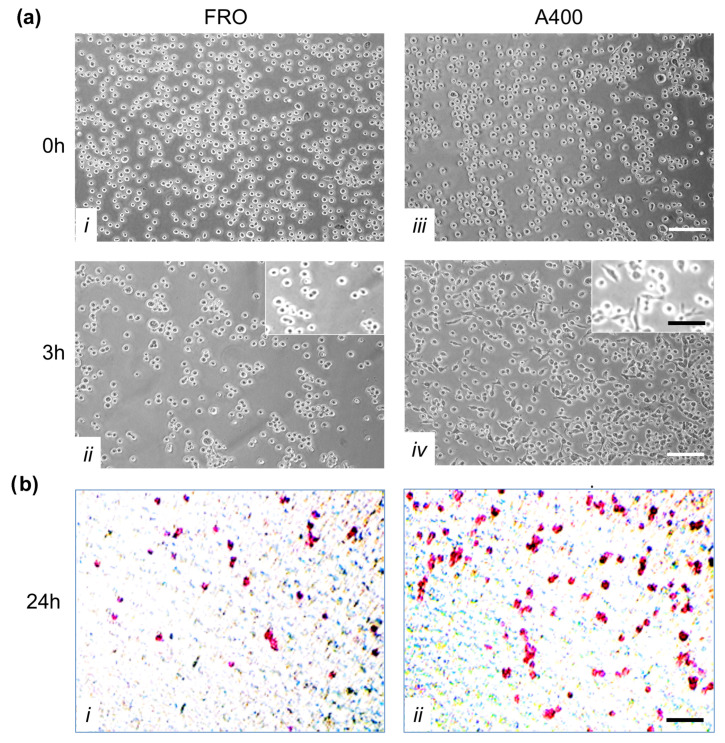
A400 cells displayed increased adhesion to extracellular matrix proteins and invasivity. Panel (**a**): Increased adhesion to extracellular matrix proteins by A400 cells; 10^5^ cells/well, pre-incubated in Tn- and vehicle-free DMEM high-glucose 10% FBS for 48 h, then plated in serum-, Tn-, and vehicle-free DMEM in 24-well plates coated with Matrigel. Cells were incubated for 3 h at 37 °C. After washes, adherent cells were photographed under phase contrast at 10× magnification (insets at 20× magnification). Panels (***i***,***ii***): FRO cells at 0 and 3 h, respectively. Panels (***iii***,***iv***): A400 cells at 0 and 3 h, respectively. Panel (**b**): Increased invasivity of A400 cells. Transwell invasion assays were performed in Boyden chambers with 8 µm pore filter inserts in 24-well plates. The filters were pre-coated with 100 µL Matrigel. Cells were pre-incubated in serum-, Tn-, vehicle-free DMEM for 48 h, and then trypsinized and transferred to the upper chamber (1 × 10^5^ cells/well). After 24 h of incubation, the filter was removed from the chamber and washed. Cells were fixed, permeabilized, and stained, as reported in the Materials and Methods section. After three washes, cells were photographed. Panel (***i***): FRO cells. Panel (***ii***): A400 cells. Scale bars: white 100 μm; black 50 μm.

A crucial feature of cancer cells is their capability to invade neighboring tissues. We studied this characteristic first by evaluating adhesion to extracellular matrix proteins. To this end, we performed an adhesion assay in which FRO and A400 cells were seeded in plates coated with Matrigel. By three hours, A400 cells attached with high efficiency, unlike FRO cells (Figure 7a, panel *iv* vs. *ii*). Moreover, by three hours, several A400 cells were already spread (inset of panel *iv*), while FRO cells were still rounded (inset of panel *ii*).

Next, we performed invasion assays on Boyden chambers coated with Matrigel. As shown in Figure 7b, A400 cells showed increased invasion capability (panels *ii* vs. *i*).

## 4. Discussion

In this study, we subjected an ATC cell line that had not undergone a full EMT to strong ER stress, such as Tn 400 ng/mL. This caused the death of a large fraction of cells, but eventually, a population emerged and, somewhat surprisingly, continuously grew in the presence of Tn 400 ng/mL. The adaptation process did not imply a simple death/life decision but was accompanied by an exacerbation of mesenchymal features with an increased invasive phenotype. Thus, at variance with the “mesenchymal shift” that we showed in normal thyroid cells following ER stress [31], we named these phenotype changes in cancer cells “mesenchymal drift”. The adapted cells showed, at the level of a single cell, caught in the act of moving, with high expression of vimentin, FN, and N-cadherin.

The TME of solid tumors plays a crucial role in cancer progression. The limited nutrient and oxygen availability activates several pathways, like the HIF pathway, which increases survival and stimulates angiogenesis and aerobic glycolysis [5,6,7]. TME causes ER stress, probably through protein misfolding, and this in turn activates the UPR [14,15]. It has been shown that cancer cells activate the PERK-eIF2α-ATF4 axis to mediate hypoxia tolerance [14], translation of angiogenic factors [48], upregulation of autophagy genes [49], and crosstalk with the HIF pathway [50,51].

A puzzling characteristic of the UPR is the simultaneous activation of survival and death pathways. Thus, efforts have been devoted to understanding the life/death switch mechanisms. It has been shown that an adaptive response is a consequence of the preferential stabilities of mRNA and proteins that facilitate adaptation (molecular chaperones) with respect to those that facilitate cell death (CHOP, GADD34) [24]. Moreover, the timing of ER stress also plays a role. Persistent, chronic ER stress activated only the PERK pathway, while it attenuated IRE signaling, favoring cell death [25]. We show here that ER stress may act as a selection mechanism, since the cell population emerging from strong ER stress acquires a growth advantage over parental cells. Thus, activation of the UPR is attenuated, (inhibited BiP induction, eIF2α phosphorylation, CHOP induction—Figure 3; inhibited activation of the stress kinases—Figure 4). The stress kinases, JNK, p38, and ERK, are targets of the UPR, downstream IRE1 [44,45,46]. Moreover, apoptosis is suppressed in A400 cells compared to FRO cells (Supplementary Figure S1). This is in line with the lack of induction of CHOP and of JNK phosphorylation, two of the major mediators of cell death following ER stress [16,29]. The final result is a survival gain of A400 cells in a harsh microenvironment.

However, data obtained by us and others [27,28,29,52,53,54], using normal and differentiated cells, suggest that what appears to be a pure binary decision may in fact be more complex. Also, a third alternative exists, in that survival may be achieved at the expense of differentiation, and in thyroid epithelial cells, a variable number of mesenchymal markers appear to differing extents [30,31]. We hypothesized that these findings may be even more significant in a cancer context, where ER stress is linked to cancer progression. We selected FRO cells to study this aspect, since they have not undergone a full EMT. FRO cells express considerable levels of E-cadherin and low levels of VIM, unlike 8505C and CAL62 cells (Figure 1). This finding is in line with those showing that FRO cells harbor the BRAF V600E mutation, but have wildtype p53, albeit under-expressed [32,39], while 8505C and CAL62 cells, in addition to an proto-oncogene mutation [33], both carry loss-of-function p53 mutations [34,38]. Thus, FRO cells represent early stages of cancer progression.

EMT is a central component of cancer progression [55]. The term EMT indicates complete trans-differentiation from the epithelial to the mesenchymal phenotype, secondary to total transcriptional reprogramming. Often, however, there are EMT-like processes that do not configure a full EMT. During tumor progression, it is more appropriate to refer to the emergence of EMT-like phenotypes, with different degrees of progression towards a full EMT [55,56,57,58].

In scratch assays, A400 cells showed enhanced capability compared to FRO cells to detach from other cells and migrate into the scratch, giving rise to a single-cell mode of migration (Figure 5). Increased expression of FN and vimentin is involved in the invasion of several tumors [59,60,61,62]. E- and N-cadherin have been shown to affect the behavior of epithelial tumor cells in a diametrically opposed way, although both mediate cell–cell adhesion in cells in which they are normally expressed. E-cadherin is expressed widely in epithelial tissues where it maintains strong cell–cell adhesion and tissue architecture [63,64]. Loss of E-cadherin mediated cell–cell adhesion facilitates the dissemination of tumor cells and confers upon them a more invasive state. N-cadherin is predominantly expressed by neuronal and mesenchymal cells [65,66], but it is often upregulated in epithelial tumors and has been shown to correlate with motility and invasiveness [64,67,68,69,70,71]. Also, several studies have shown that one of the hallmarks of tumor progression and EMT is the gain of N-cadherin expression by tumor cells, which may or may not be accompanied by the concomitant loss of E-cadherin expression, a phenomenon commonly referred to as cadherin switching [58,63,64,67].

The double immunofluorescence experiments show a prevalent overlap between the N-cadherin and β-catenin signal in A400 cells, with respect to the E-cadherin–β-catenin overlap (Supplemental Figure S2), suggesting a cadherin switch based on cadherin cellular dynamics. To pursue this suggestion, we set up an original assay, a scratchwound migration assay coupled to immunofluorescence staining. Evidently, this assay gives neither global expression levels nor cellular localization (as in Figure S2) in a static cell population but is able to show the localization of mesenchymal proteins in cells caught in the act of moving, following a dynamic trigger (the scratch). The singly moving A400 cells and those located at the front were heavily stained with vimentin, FN, and N-cadherin and showed evident lamellipodia in the direction of the movement. On the contrary, FRO cells showed a dramatic decreased tendency to move into the scratchand were less stained with VIM, FN, and N-cadherin (Figure 5 and Figure 6).

As noted above, EMT-like phenotypes of cancer cells simultaneously display mesenchymal and epithelial characteristics [55,56,57]. This state, known as the intermediate or metastable phenotype, is believed to be a crucial factor for full metastatic competence, allowing cancer cells to dynamically adjust to the circumstances they encounter [72,73,74,75]. Our data suggest that cells selected and adapted to ER stress may reproduce a metastable phenotype, in that they dynamically increase the expression and, possibly, cellular localization of mesenchymal proteins while moving in appropriate conditions (following a scratch), although we cannot say if these phenotypic changes were the cause that initiated the movement or the consequence of it.

Moreover, these data are reminiscent of in vivo data, in which the emergence of EMT-like phenotypes in carcinoma cells occurs typically at the invasive front of primary tumors. These cells are considered to be those that eventually give rise to the subsequent steps of the invasion–metastasis cascade [67,72,76,77,78].

## 5. Conclusions

Excessive or prolonged ER stress is believed to end with apoptotic cell death [24,25,26]. Here, we show that strong ER stress acts as a selection factor, favoring the emergence of a cell population, showing exacerbation of the malignant phenotype. The selected cell population shows increased single-cell motility, invasion, and high mesenchymal protein expression in the moving component, which has lost cell–cell contact. These features may be defined as a “mesenchymal drift”, reproducing, in a cancer context, what we have observed in a normal thyroid cell population [31] and thus previously called a “mesenchymal shift”.

## Data Availability

The original contributions presented in this study are included in the article and Supplementary Material. Further inquiries can be directed to the corresponding authors.

## Supplementary Materials

The following supporting information can be downloaded at https://www.mdpi.com/article/10.3390/cancers17213534/s1: Figure S1: Apoptosis is suppressed in A400 cells; Figure S2: Cellular distribution of E-cadherin, N-cadherin, and β-catenin; Figure S3: FRO cells have not undergone full EMT; Figure S4: UPR signaling is attenuated in A400 cells; Figure S5: Densitometric analysis of BiP/actin, CHOP/actin, and P-eIF2α/eIF2α; Figure S6: The activation of stress kinases is attenuated in A400 cells; Figure S7: Densitometric analysis of P-ERK/ERK, P-JNK/JNK, P-p38/p-38; Figure S8: Apoptosis is suppressed in A400 cells.
