# Supplementary material for: ER Stress Is Associated with a “Mesenchymal Drift” in an Anaplastic Thyroid Carcinoma Cell Line
Source: Cancers (Basel). 2025 Oct 31;17(21):3534. doi: 10.3390/cancers17213534 (PMC12610349; doi:10.3390/cancers17213534)
Supplement: Supplementary file 1 [file cancers-17-03534-s001.zip › cancers-3713632-supplementary.pdf]

cancers

MDPI

Supplementary Materials

ER Stress Is Associated with a "Mesenchymal Drift" in an Anaplastic Thyroid Carcinoma Cell Line

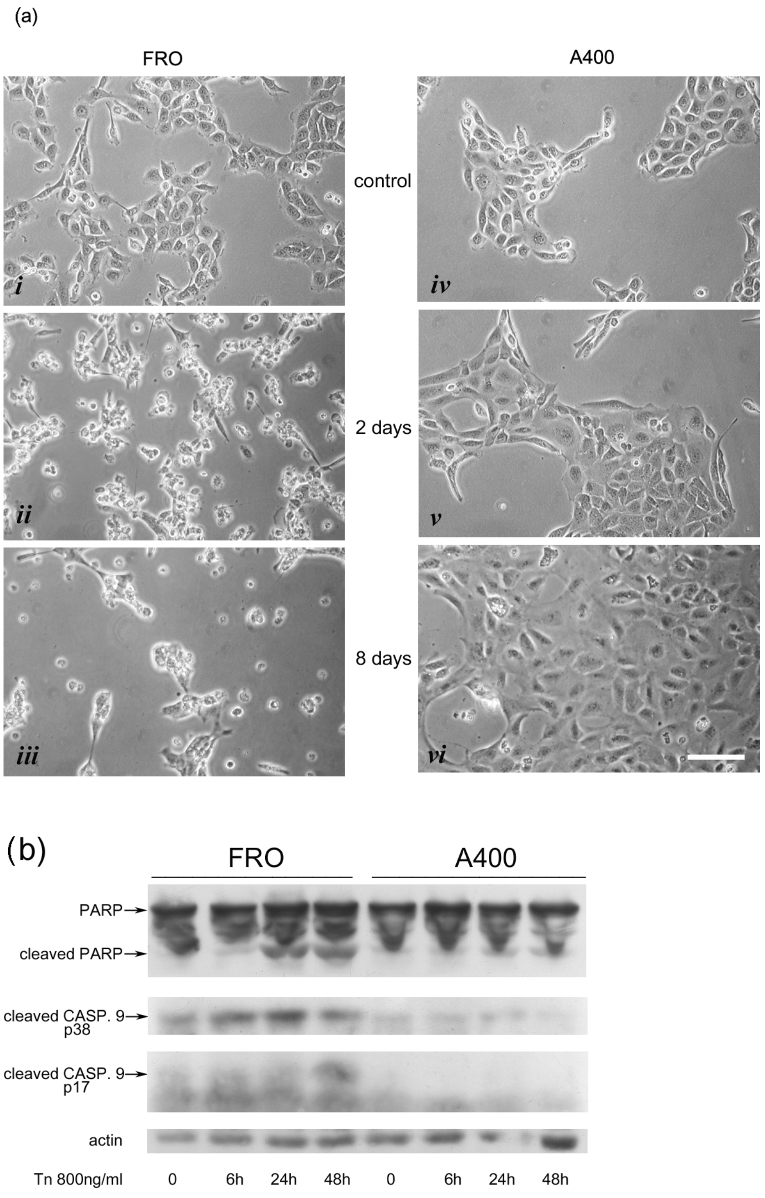

Figure S1. Apoptosis is suppressed in A400 cells. Cells were plated in 60 mm diameter plates at about 50% confluence in DMEM high glucose 10% FBS with Tn (400 ng/ml, A400 cells) or vehicle (FRO cells). After 24 h, Tn and vehicle (in A400 and FRO cells, respectively) were removed from the culture medium for 48 h. Then, 800 ng/mL Tn was added for various times to both cell lines. Panel a, cells were photographed at time 0, day 2, and day 8. Scale bar: 50 m Panel b, Western blots of total protein extracts from FRO, and A400 cells were made at the indicated times as indicated in Materials and Methods. A400 cells show cleavage of the general apoptosis marker PARP and activation of caspase 9 (CASP. 9), at variance with FRO cells. Notably, cleaved PARP and caspase activation peaked at 24-48 hrs, synchronously with CHOP (Fig. 3A), that is a crucial apoptosis inducer of the UPR (Sano and Reed, 2013). Note that the anti-caspase 9 used (Cell Signaling #9507) detects endogenous levels of the large fragment (17 kDa and 38 kDa with prodomain) of caspase 9 resulting

from cleavage at aspartic acid 353. The antibody does not recognize full length caspase 9. Uncropped western blot images are shown in Supplementary Material Figure S8.

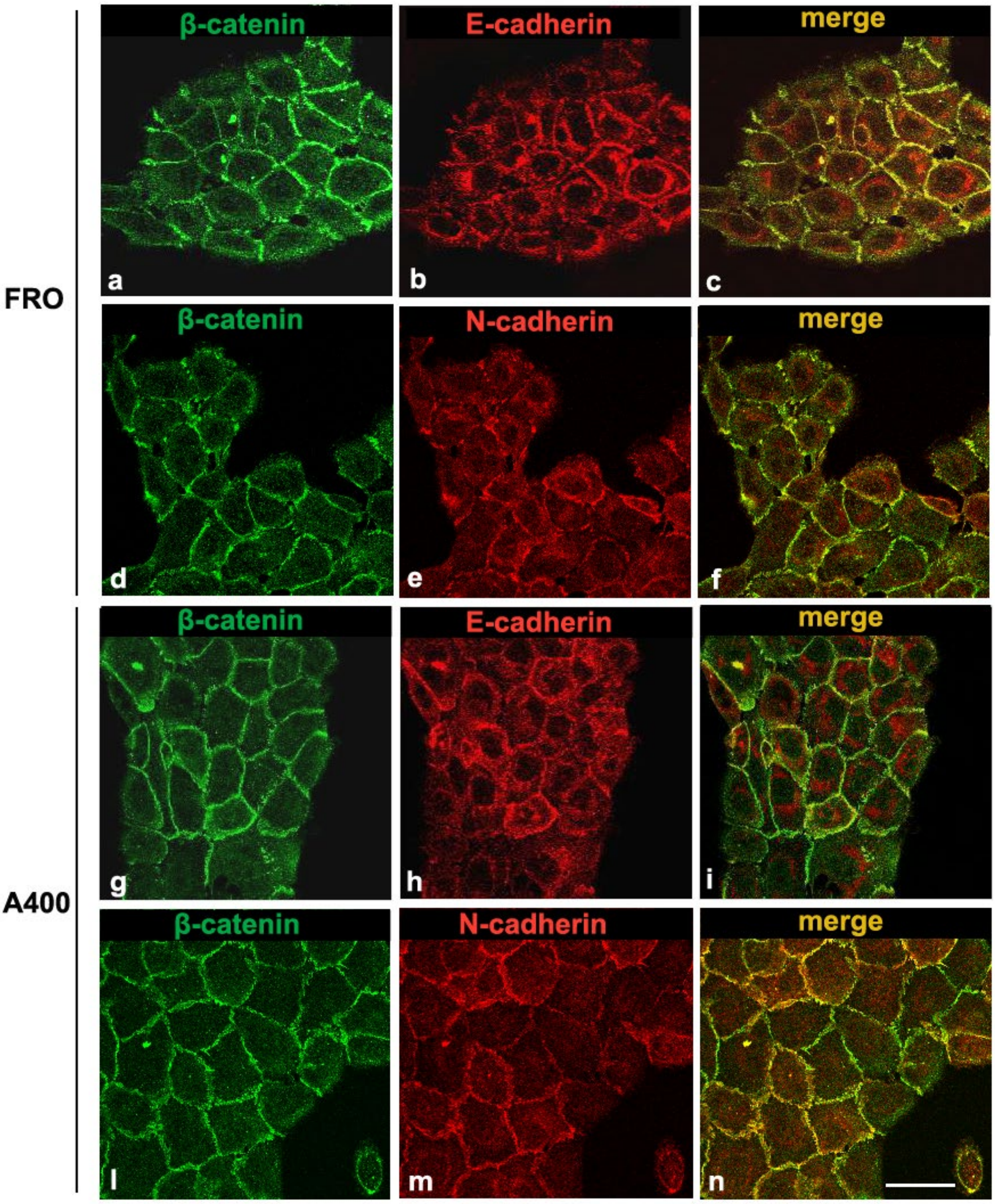

Figure S2. Cellular distribution of E-cadherin, N-cadherin, and β-catenin. In FRO cells, the E-cadherin/ -catenin overlap at the plasmamembrane dominates over the N-cadherin/ -catenin overlap. In A400 cells, reciprocally, the N-cadherin/ -catenin overlap at the plasmamembrane dominates over the E-cadherin/ -catenin overlap. Cells were plated on glass coverslips and grown to sub-confluence in DMEM high glucose 10% FBS with Tn (A400 cells) or vehicle (FRO cells). Then, cells were incubated in DMEM without Tn or vehicle for 48 hours, and processed for immunofluorescence as indicated in Materials and Methods. Panels a-f FRO cells; panels g-n A400 cells. Scale bar: 20 m.

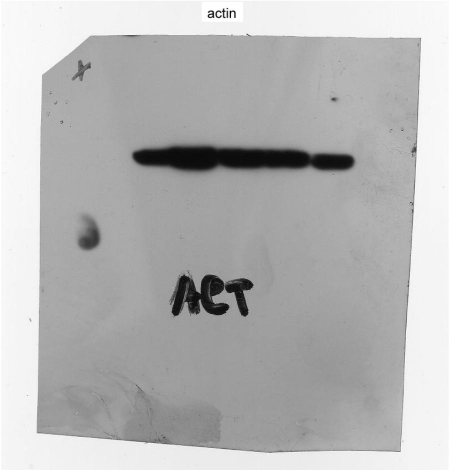

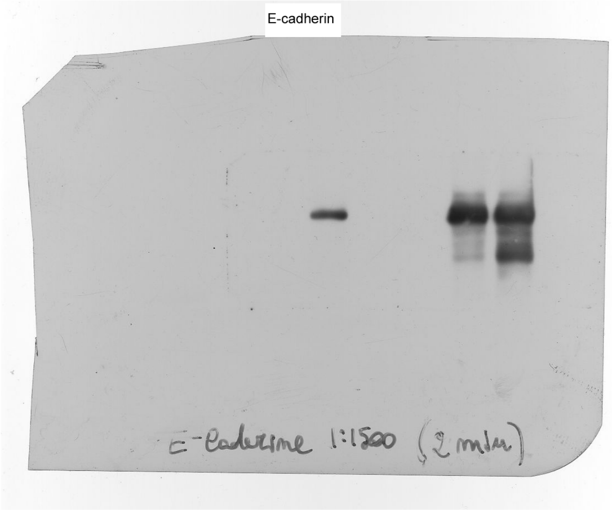

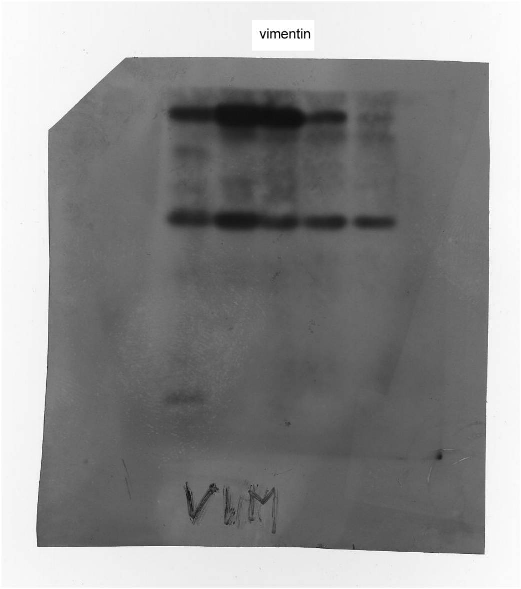

Figure S3. FRO cells have not undergone full EMT

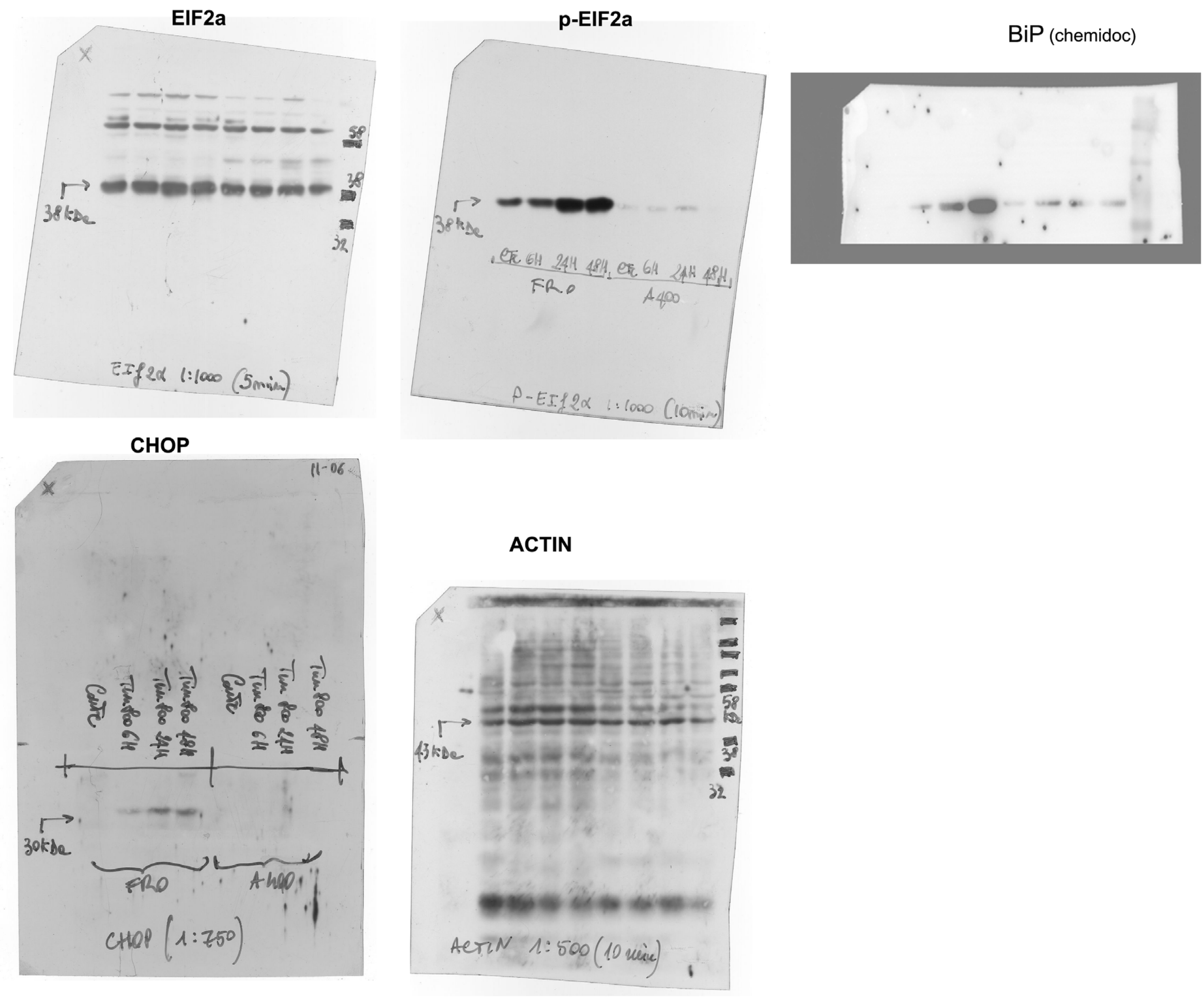

Figure S4. UPR signaling is attenuated in A400 cells.

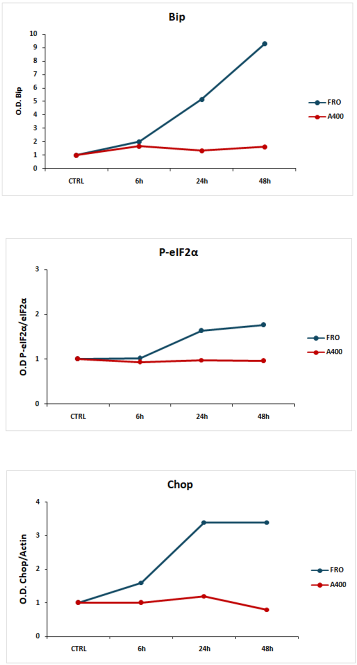

Figure S5. Densitometric analysis of BiP/actin, CHOP/actin, and P-eIF2α/eIF2α.

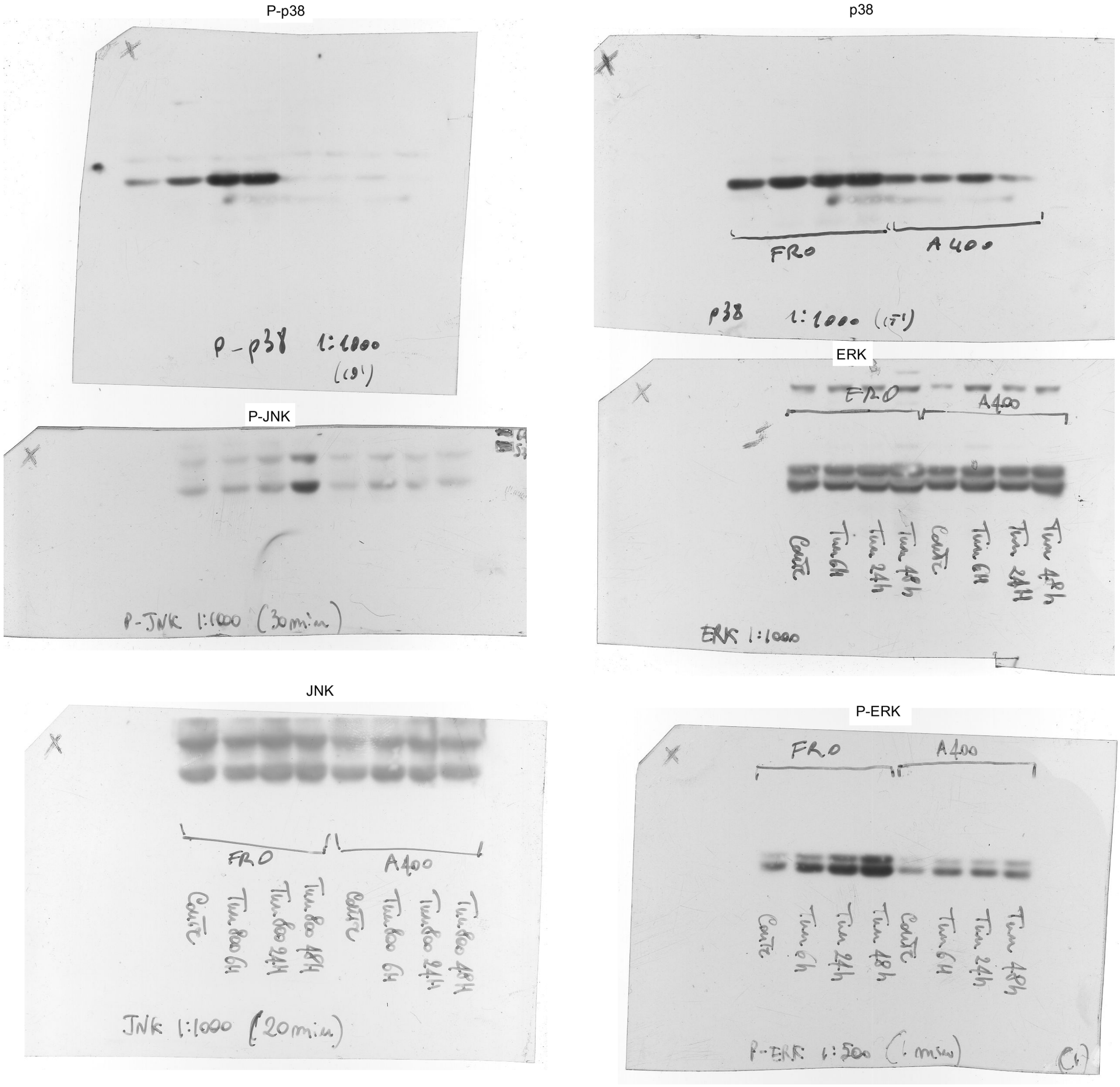

Figure S6. The activation of stress kinases is attenuated in A400 cells.

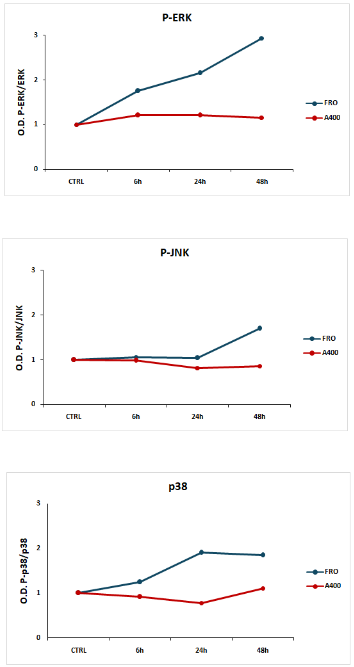

Figure S7. Densitometric analysis of P-ERK/ERK, P-JNK/JNK, P-p38/p-38.

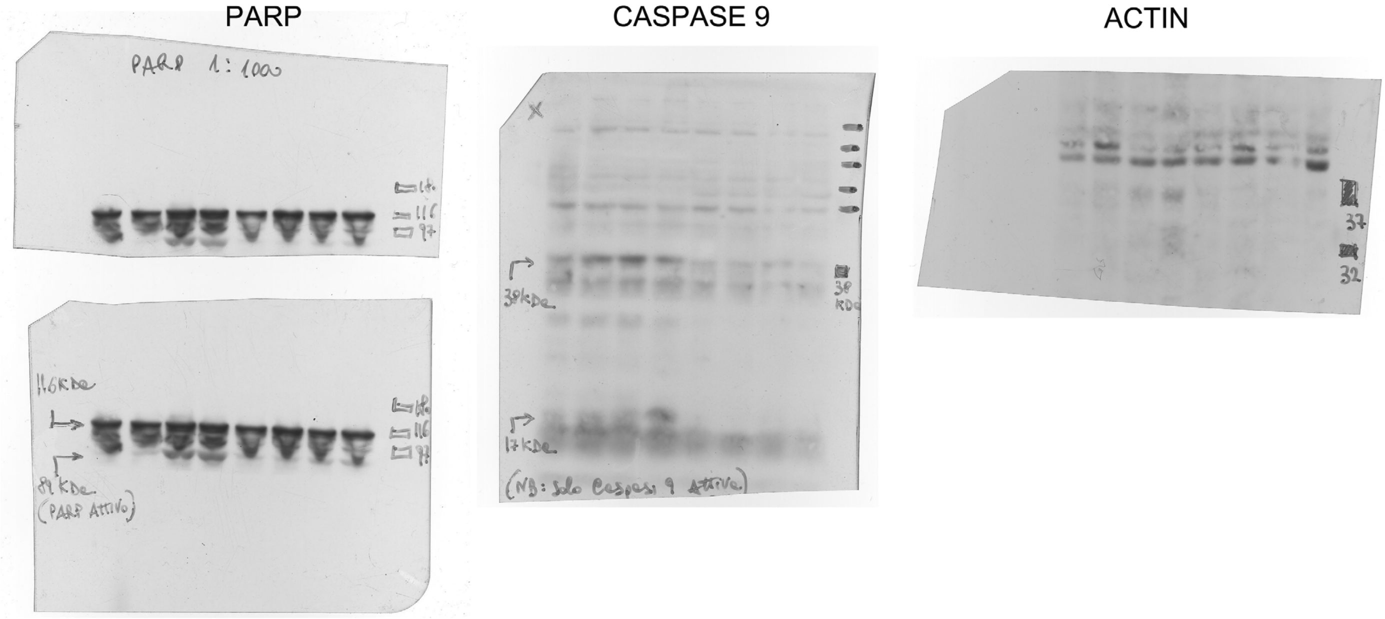

Figure S8. Apoptosis is suppressed in A400 cells.
